# Age-related differences in Rostral-Middle locus coeruleus microstructure: A critical role in cognitive decline revealed by magnetic resonance relaxometry

**DOI:** 10.1186/s13195-025-01809-4

**Published:** 2025-07-15

**Authors:** Jonghyun Bae, Zhaoyuan Gong, Caio Mazucanti, Murat Bilgel, John P. Laporte, Mary E. Faulkner, Alex Guo, Christopher M. Bergeron, Josephine M. Egan, Susan M. Resnick, Christopher E. Ramsden, Mustapha Bouhrara

**Affiliations:** 1 Laboratory of Clinical Investigation, National Institute on Aging, National Institutes of Health, Baltimore, MD 21224 USA; 2 Laboratory of Behavioral Neuroscience, National Institute on Aging, National Institutes of Health, Baltimore, MD 21224 USA; 3 Magnetic Resonance Physics of Aging and Dementia Unit, Laboratory of Clinical Investigation, National Institute on Aging (NIA), NIH, BRC 05C-222, 251 Bayview Blvd, Baltimore, MD BRC 05C-222, 21224 USA

**Keywords:** Locus coeruleus, MRI biomarker, Relaxometry, Aging, Cognition

## Abstract

**Background:**

The Locus Coeruleus (LC) is a critical brain region affected by neurodegenerative diseases and aging. Despite its importance, in-vivo investigations of age-related LC degeneration and association with cognitive decline have been limited.

**Method:**

We employed magnetic resonance relaxometry, namely the Bayesian Monte-Carlo analysis of multicomponent driven equilibrium single pulse observation of T_1_ and T_2_ (BMC-mcDESPOT) MRI method, to estimate microstructural integrity represented by longitudinal (R_1_) and transverse (R_2_) relaxation rates, as well as Myelin Water Fraction (MWF) in the LC of a diverse cohort of 120 cognitively unimpaired individuals aged 22 to 94 years. BMC-mcDESPOT offers high spatial resolution and is effective for mapping detailed microstructural changes within the LC. We examined age-related differences in LC microstructure, their associations with cognitive changes, and the spatial variation of these microstructural changes within the LC, exploring their distinctive contributions to cognitive decline.

**Results:**

LC-R_2_ values declined significantly with age, particularly in the rostral-middle regions. LC-R_1_ and LC-MWF values showed significant positive correlations with cross-sectional memory scores. Longitudinally, the rostra-middle LC-R_2_ values showed an age-moderated effect, with lower values predicting steeper memory decline at advanced ages.

**Conclusions:**

Quantitative MR relaxometry reveals that LC microstructural integrity declines with age and is predictive of cognitive decline, particularly in memory. Our MR relaxometry biomarkers, especially in the rostral LC, serve as sensitive imaging biomarkers of early structural alterations and cognitive declines in aging.

**Supplementary Information:**

The online version contains supplementary material available at 10.1186/s13195-025-01809-4.

## Background

The locus coeruleus (LC), a nucleus in the rostral pontine brainstem (14.5 mm in length and 2.5 mm in thickness on average) [1], plays a crucial role in the brain’s noradrenergic system. The LC’s noradrenergic neurons project to cortical and subcortical regions, modulating sleep and wake cycles [2], memory [3], and attention [4]. LC degeneration occurs in neurodegenerative diseases, such as Alzheimer’s disease (AD). Previous preclinical studies have linked LC impairments to amyloid pathology, neuroinflammation, and neurovascular dysfunction [5]. Moreover, LC degeneration is more strongly associated with tau pathology, with evidence suggesting that the first alterations in tau, such as phosphorylated tau accumulation, occur in the LC [6]. Additionally, a previous study found tau lesions in the LC in 94% of individuals by the age of 50, highlighting the prevalence of tau pathology [7]. Further, several studies [2, 8, 9] suggest spatially differentiated LC structure, where rostral LC neurons make projections to hippocampal regions and modulate cognitive functions, such as learning and memory, by releasing norepinephrine (NE) [10–12]. In contrast, caudal regions of LC neurons innervate the cerebellum and spinal cord [13, 14]. Based on findings that highlight the crucial role of the LC in age-related cognitive decline [15, 16] and its implications in the progression to neurodegenerative diseases [17], it is essential to develop in vivo biomarkers that accurately reflect topographical microstructural changes in LC integrity. Although several MRI studies have attempted to assess LC integrity, these methods often lack specificity and are less quantitative. We therefore aim to fill this deficit and apply state-of-the-art MR imaging of LC.

Previous MRI studies have identified differences in LC structure using T_1_-weighted images obtained with turbo spin echo sequences [18]. The neuromelanin-rich LC neurons exhibit a T_1_-shortening effect, appearing hyperintense [19]. Most studies assess LC integrity by calculating the contrast ratio (CR) between LC signal intensity and a reference region [8, 20–23], while a few have attempted to measure volumetric changes [24]. Although a previous study [25] reported the association of reduced CR and volume with indicators of AD pathology, other studies present conflicting results [26, 27]. Similarly, studies on LC changes during normal aging have yielded inconsistent results [28, 29]. Moreover, these MRI studies mostly employ 2D imaging approach with thick slices of ~ 3 mm, potentially hindering accurate assessment in topographical changes of LC structure. Despite their potential for monitoring aging processes and pathological development, there is an unmet need for quantitative metrics to assess LC microstructural integrity and its impact on cognitive functioning. Microstructural changes have been shown to precede, by decades, macrostructural changes [30]. In addition to establishing imaging biomarkers for early detection of abnormal changes in LC microstructure, such investigations in microstructural changes are critical to decipher the mechanisms underlying structural changes in the LC and to differentiate those due to normal from abnormal aging. The paucity of such studies is largely due to the small size of LC. Therefore, it is essential to use high spatial resolution imaging to minimize partial volume effects.

Despite its’ effectiveness in probing tissue microstructure and composition of the brain [31], the use of quantitative MR relaxometry for evaluating the LC's structural integrity has been limited. Recently, a handful of studies explored multi-parametric mapping approaches incorporating quantitative metrics, such as longitudinal relaxation rate (R_1_ = 1/T_1_), and/or Magnetic Transfer saturation (MT-sat). Wearn et al. [32] evaluated multiple MRI biomarkers in the LC, including R_1_, R_2_^*^ (apparent transverse relaxation rate), MT-sat, and Proton Density, in relation to CSF biomarkers of AD pathology, but did not examine cognition. Turner et al. [33] focused on MT-sat to assess LC integrity and provided evidence linking reduced LC integrity in older adults to a decision-making bias, suggesting that LC integrity is crucial for maintaining adaptive cognitive behavior in aging. These studies highlight the potential of quantitative imaging but show that relaxometry biomarkers remain underexplored. Moreover, a previous study [34] failed to detect LC contrast in R_1_ in a limited number of participants, yet another study [35] argues that the observed contrast in T_1_- and Magnetization Transfer (MT)-weighted scans is primarily driven by R_1_ effect. As demonstrated in previous MRI studies, neuromelanin– a by-product of catecholamines– is paramagnetic and shortens longitudinal relaxation time (T_1_ = 1/R_1_). Therefore, a reduction in R_1_ may indicate alterations in the proper functioning of the noradrenergic system. Additionally, transverse relaxation time (T_2_ = 1/R_2_) derived from MR relaxometry may reflect microstructural integrity, such as cellular density, as reflected by a high correlation with apparent diffusion coefficient (ADC) [36]. Finally, although both histological evidence [35] and age-related changes in MT-sat measures [33] suggest low but detectable myelin content in the LC, this aspect remains largely unexplored. In addition to R_1_ and R_2_, myelin content can serve as a complementary metric for assessing the microstructural integrity of the LC. To evaluate the structural integrity of the LC, we employed the Bayesian Monte-Carlo analysis of multicomponent driven equilibrium single pulse observation of T_1_ and T_2_ (BMC-mcDESPOT) MRI method [37] for high spatial resolution mapping of relaxation rates (R_1_, R_2_) and Myelin Water Fraction (MWF) [37, 38]. This quantitative MRI (qMRI) technique has been adopted in several clinical investigations of central nervous system maturation [39] and degeneration [39–41].

Our study cohort consisted of 120 cognitively unimpaired participants spanning a well-characterized age range of 22 to 94 years. BMC-mcDESPOT has been used extensively to investigate the patterns of cerebrum and brainstem myelination, composition and microstructural changes with aging and dementias [40, 42]. Our main goals are; *i*) to investigate age-related differences in LC microstructure, and *ii*) to assess the association of differences in LC microstructure with changes in cognition. We herein test the hypotheses that; *i*) LC’s microstructural integrity is lower at advanced ages, and *ii*) lower microstructural integrity of LC is associated with lower memory retention and attention, and with their steeper declines over time. This study aims to deepen our understanding of the relationship between LC’s structural integrity with age and cognition. This knowledge may allow for developing imaging biomarkers sensitive to early changes in LC to help monitor targeted interventions in the brain.

## Methods

### Participants

The study sample comprised participants drawn from two National Institute on Aging / National Institutes of Health Intramural Research Program (NIA/NIH IRP)-approved studies: the Baltimore Longitudinal Study of Aging (BLSA; Protocol #03AG0325) [43] and the Genetic and Epigenetic Signatures of Translational Aging Laboratory Testing (GESTALT; Protocol #15AG0063). Both studies adhered to identical inclusion and exclusion criteria, with a shared objective of assessing multiple aging-related biomarkers. Prior to enrollment in our MRI protocol, stringent eligibility criteria were applied, ensuring the absence of central nervous system diseases (e.g., dementia, stroke, bipolar illness, epilepsy), cardiac disease, pulmonary disease, and metastatic cancer. Participants with metallic implants or significant neurological or medical disorders were excluded from the MRI cohort. Participants were determined to be cognitively normal if they had ≤ 3 errors on the Blessed Information-Memory-Concentration (BIMC) Test [44], and where available, a Clinical Dementia Rating (CDR) [45] of zero. If participants had > 3 errors on the BIMC or a CDR > 0, their clinical and neuropsychological data were thoroughly reviewed as part of consensus case conferencing. Visits where participants were found to have cognitive impairment or dementia were excluded from these analyses.

**Fig. 1:**
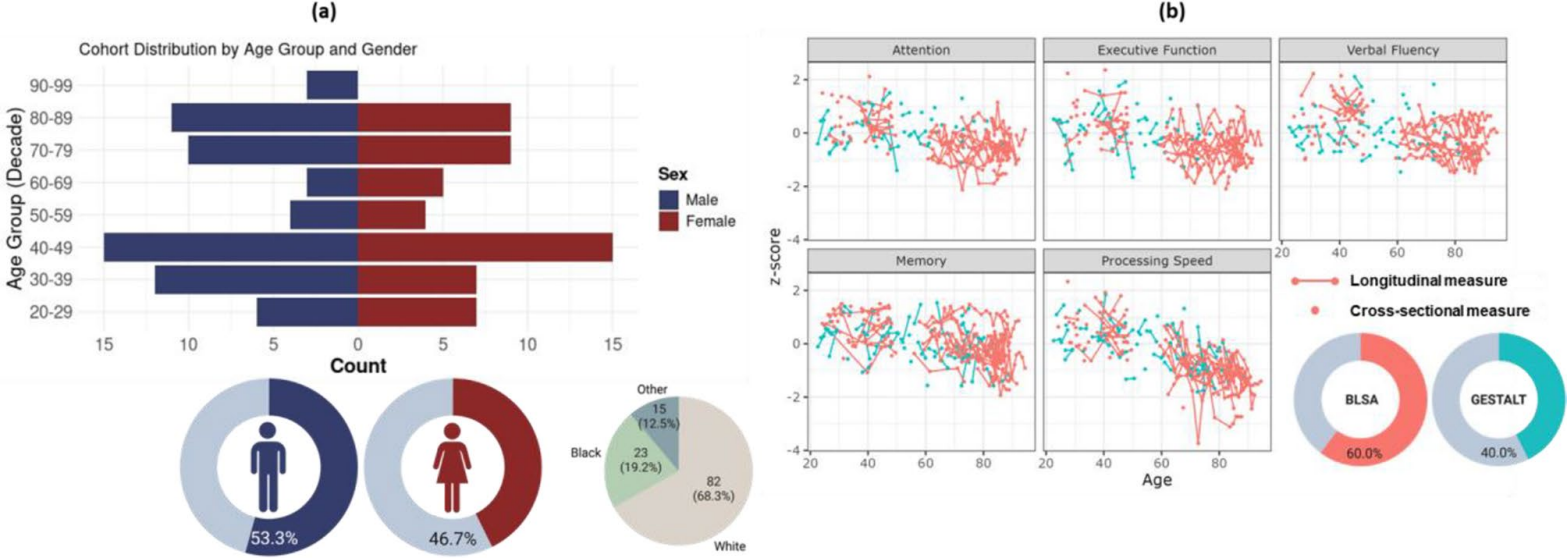
(**a**)Participants' distribution by age group, sed and race. Our study included participants aged 22 and 94 years, with a well-balanced distribution between the sexes and a fair representation of both young and old age groups. (**b**)Longitudinal cognitive trajectories across multiple domains. This graphical representation displays z-scored measures for various cognitive domains, with each panel showcasing a different domain. Longitudinal cognitive data for each participant is represented by a dot and connected lines, illustrating individual trajectories over time. The cohorts are distinguished by color, with red indicating participants from the Baltimore Longitudinal Study of Aging (BLSA) and blue representing those from the Genetic and Epigenetic Signatures of Translational Aging Laboratory Testing (GESTALT) study

### Neuropsychological assessment

Cognitive domain scores were obtained for memory (California Verbal Learning Test [46] immediate and long-delay free recall), attention (Trail Making Test Part A (TMT A) [47] and Digit Span [48] Forward), executive function (Trail Making Test Part B and Digit Span Backward), verbal fluency (Category [49] and Letter Fluency [50]) and processing speed (Digital Symbol Substitution Test (DSST) [51]). Each test score was first converted to a z-score using the baseline mean and standard deviation. When multiple tests were administered within a cognitive domain, these z-score were averaged to compute the domain score. For processing speed, we adopted a composite score as suggested before [52], and combined results from TMT A and DSST. We note that individual scores for Trail making Test Parts A and B were log-transformed and scale-inverted prior to computing z-scores, so that all high z-scores reflect shorter completion times. Figure 1b demonstrates the distribution of test scores against participant’s age for each cognitive domain. Most participants (63%) underwent cognitive evaluation for memory at least twice at or before the MRI scan. For other cognitive domains, 52% of participants had at least two cognitive evaluations before or at the time of MRI scan.

### MRI acquisition

All MR scans were acquired on a 3T whole-body Philips MRI system (Achieva, Best, The Netherlands) using the internal quadrature body coil for radio-frequency (RF) transmission and an eight-channel phased-array head coil for reception. Each participant underwent our BMC-mcDESPOT protocol [37], consisting of 3D spoiled gradient echo (SPGR) sequences (TE/TR = 1.48/5 ms) and balanced steady state free precession (bSSFP) sequences (TE/TR = 2.8/5.9 ms) with RF excitation pulse phase increment of 0 and $\:\pi\:$ to account for off-resonance artifacts [53]. All three sequences were acquired with multiple flip angles: (2, 4, 6, 8, 10, 12, 14, 16, 18, 20)$\:^\circ\:$ for SPGR and (2, 4, 7, 11, 16, 24, 32, 40, 50, 60)$\:^\circ\:$ for bSSFP sequences. All images were acquired with acquisition matrix of $\:150\times\:130\times\:94$ and an isotropic voxel size of 1.6 mm, reconstructed to a 1 mm isotropic resolution. To correct the RF inhomogeneity, $\:{B}_{1}$, the double-angle method [54] was implemented with two fast spin-echo images acquired with FAs of 45$\:^\circ\:$ and 90$\:^\circ\:$ (TE/TR = 102/3000 ms, spatial resolution = $\:2.6\times\:2.6\times\:4\:mm$). Estimated low-resolution $\:{B}_{1}$ field was further interpolated to align with the same spatial resolution of the SPGR and bSSFP images.

### Data processing and region segmentation

For each participant, a whole-brain $\:{R}_{1}$ map was generated from the SPGR and DAM datasets using DESPOT1 and assumes a single component, and a whole-brain $\:{R}_{2}$ map was generated from the bSSFP and DAM datasets using DESPOT2 assuming a single component [55]. Finally, MWF was estimated, assuming a two-component non-exchanging system consisting of short and long T_1_ and T_2_ components. The short component corresponds to the signal of water trapped within the myelin sheets, while the long component corresponds to intra/extra-cellular water. The averaged SPGR image for each case was registered to the Montreal Neurological Institute (MNI) template (MNI-ICBM 152 linear space, 0.5 mm resolution), provided by a previous study (See [8] for the details on standardization). We used the Automated Brainstem Co-registration (ABC) method [56], which involves an initial global registration between individual SPGR images and the MNI template image, followed by a brainstem-weighted registration with dilated brainstem mask from FreeSurfer [57]. The registration settings were adopted as described in the original publication. Using the resulting transformation matrix, individual $\:{R}_{1}$,$\:{R}_{2}$ and MWF maps were then warped to the template space. For LC localization, we adopted a meta-mask provided by Dahl et al. [58], which combines previously reported LC masks to enhance the identification of small LC structures. Using the meta-mask, we calculated the mean values of qMRI metrics ($\:{R}_{1}$, $\:{R}_{2}$ and MWF) across the entire LC structure for each participant. To achieve topographical assessment of LC, we computed slice-averaged qMRI values. Then, we divided the LC along its rostrocaudal axis into three equidistant segments, following a previous study [59], and defined rostral-middle (0–66th LC rostro-caudal percentile) and caudal (66–100th LC rostro-caudal percentile) regions of the LC. Additionally, we evaluated the mean values of qMRI measures for both the rostral-middle LC and caudal regions of the LC.

### Statistical analysis

#### Age-related differences in the LC microstructural integrity

We first examined differences in LC microstructure or myelination with aging. Each of the qMRI measures was correlated with age while controlling for the effects of sex, years of education (EDY) and race using the following multiple linear regression model.

1 $$\begin{aligned}qMR{I_i} & = {\beta _0} + {\beta _{age}} \times ag{e_i} \\&\quad + {\beta _{sex}} \times se{x_i} \\&\quad + {\beta _{race}} \times rac{e_i} \\&\quad + {\beta _{EDY}} \times ED{Y_i} + {\varepsilon _i} \\&\end{aligned} $$

where $\:q{MRI}_{i}$ is qMRI metrics (R_1,_ R_2_ or MWF), $\:{age}_{i}$ is age of the *i*^*th*^ participant at the time of MRI, and $\:{\epsilon\:}_{i}\:$is the residual.

We also examined non-linear trends in changes to LC integrity, as suggested by other studies [60]. The non-linear model accounting for potential, quadratic, inverted U-shaped trends is given by:

2 $$\begin{aligned}\:{qMRI}_{i}&=\:{\beta\:}_{0}+{\beta\:}_{age}\times\:{age}_{i}\cr&\quad+{\beta\:}_{{age}^{2}}\times\:{{age}_{i}}^{2}\cr&\quad+{\beta\:}_{sex}\times\:{sex}_{i}\cr&\quad+{\beta\:}_{race}\times\:{race}_{i}\cr&\quad+{\beta\:}_{EDY}\times\:{EDY}_{i}+{\epsilon\:}_{i},\end{aligned}$$

where $\:{{age}_{i}}^{2}$ is age-squared of the *i*^*th*^ participant at the time of MRI, and $\:{\epsilon\:}_{i}\:$is the residual. Age was mean centered to avoid collinearity between the age and the age^2^ terms.

#### Association between LC microstructural integrity and cognition

Then we conducted a cross-sectional analysis of the association between each of the qMRI measures and each of the five cognitive domains. The dependent variable was each of the z-scored cognitive domain at the time of MRI while the independent variables were age at the time of MRI, sex, race, EDY and qMRI using the following multiple linear regression model.

3 $$\begin{aligned}\:{Cog}_{i}&=\:{\beta\:}_{0}+{\beta\:}_{age}\times\:{age}_{i}\cr&\quad+{\beta\:}_{sex}\times\:{sex}_{i}+{\beta\:}_{race}\times\:{race}_{i}\cr&\quad+{\beta\:}_{EDY}\times\:{EDY}_{i}\cr&\quad+{\beta\:}_{qMRI}\times\:{qMRI}_{i}+{\epsilon\:}_{i},\end{aligned}$$

where $\:{Cog}_{i}$ is the cognitive domain score at the time of MRI, while the other terms are defined above.

#### Association between LC microstructural integrity and changes in cognition

We investigated the association between each of the qMRI measures and longitudinal changes in cognition as well as the moderating effect of age on this relationship. We employed separate linear mixed-effects models for each cognitive domain. The longitudinal cognitive scores were considered as the dependent variables while age at MRI, sex, race, EDY, time, qMRI measures, and their three-way interaction (age $\:\times\:\:$qMRI$\:\:\times\:$ time), including all lower-order terms, were the independent variables. The linear mixed effect model for the longitudinal analysis is given by

4 $$\begin{aligned}\:{Cog}_{ij}&=\:{\beta\:}_{0}+{\beta\:}_{age}\times\:{age}_{i}\cr&\quad+{\beta\:}_{sex}\times\:{sex}_{i}+{\beta\:}_{race}\times\:{race}_{i}\cr&\quad+{\beta\:}_{EDY}\times\:{EDY}_{i}+{\beta\:}_{time}\cr&\quad\times\:{time}_{ij}+{\beta\:}_{qMRI}\times\:{qMRI}_{i}\cr&\quad+{\beta\:}_{age\times\:time}\times\:{age}_{i}\times\:{time}_{ij}\cr&\quad+{\beta\:}_{qMRI\times\:age}\times\:{qMRI}_{i}\times\:{age}_{i}\cr&\quad+{\beta\:}_{qMRI\times\:time}\times\:{qMRI}_{i}\times\:{time}_{ij}\cr&\quad+{\beta\:}_{qMRI\times\:time\times\:age}\times\:{qMRI}_{i}\cr&\quad\times\:{time}_{ij}\times\:{age}_{i}+{b}_{i}+{\epsilon\:}_{ij}\end{aligned}$$

where $\:{Cog}_{ij}$ is the cognitive score of subjects $\:i$ at time $\:j$, $\:{time}_{ij}$ is the time from MRI of the *i*^*th*^ participant at time point $\:j$, and $\:{b}_{i}$ is the corresponding random intercept. To facilitate interpretation, the qMRI values were mean-centered. We note that the main parameter of interest in this analysis is $\:{\beta\:}_{MRI\times\:time}$, reflecting the expectation of the difference in the annual change in cognition per unit difference in qMRI measures, and $\:{\beta\:}_{MRI\times\:time\times\:age}$, which captures age-moderated differences in the annual rate of cognitive changes per unit difference in qMRI measures.

#### Topographical differences in LC integrity between rostral-middle and caudal regions across aging

After computing the mean values of qMRI measures for each slice, we regressed slice-averaged values on age to assess age-related topographical differences in LC integrity, following the approach described in Eq. (1). Using the estimated regression models at each slice level, we predicted qMRI values at ages 40 and 75—corresponding to the 25th and 75th percentiles of our cohort, respectively. We then examined the association between subregion-averaged qMRI measures (rostral-middle and caudal) and cognition. Similar to the models described in Eqs. (3) - (4), we assessed both cross-sectional and longitudinal cognitive outcomes, using separate models for the rostral-middle and caudal qMRI measures. Again, our primary focus was the interaction terms ($\:{\beta\:}_{MRI\times\:time}$ and $\:{\beta\:}_{MRI\times\:time\times\:age}$), which reflect whether qMRI measures in each subregion are associated with changes in cognitive function. Each qMRI metric was corrected for False Discovery Rate (FDR) across subregions (e.g. LC-R_1_, Rostral-R_1_, Caudal-R_1_) and five cognitive domains for each observed effect, using Benjamini-Hochberg procedure [61].

### Sensitivity analysis

Given the importance of accurately localizing the small LC structure, we conducted a sensitivity analysis to ensure that our observations of the association described above are specific to the LC structure rather than the surrounding tissue. For that, we dilated the LC meta-mask by 3 voxels and masked out the LC region (red mask in Figure S1b). Additionally, we deliberately shifted the LC meta-mask 1.5 mm (3 voxels in MNI space) to the left and right (red masks in Figures S1c-d), simulating a one voxel shift in our native image space while avoiding any overlap with the original mask. We sampled our qMRI measures using the shifted masks and repeated the same analysis as previously described. 

## Results

**Table 1:** Participant demographic information

Characteristics	Cognitively normal participants (*n* = 120)
Age at MRI scan, mean (SD)	55.9 (20.8)
Sex, Men (%)	64 (53.3%)
Race, White (%)	82 (68.3%)
Race, Black (%)	23 (19.2%)
Education (yrs), mean (SD)	16.3 (2.7)
Number of cognitive assess., mean (SD)	2.96 (3.3)
Duration of cognitive assess., median (mean, SD)	2.1 (4.3, 6.4)

Table 1 shows demographic information of our study cohort. Our cohort consisted of 120 subjects spanning the age range of 22 to 94 years. The participants were well-balanced in terms of sex distribution (53% male), and there were no significant differences in age between males and females (*p* > 0.1). Racial distribution was predominantly White (68%) followed by Black (19%). Participants had an average of 16 years of education. As shown in Fig. 1b, 93% of the participants had MRI scans within 2 years of the last neuropsychological assessment with only one participant undergoing the MRI scan 3 years after the last neuropsychological testing.

**Fig. 2:**
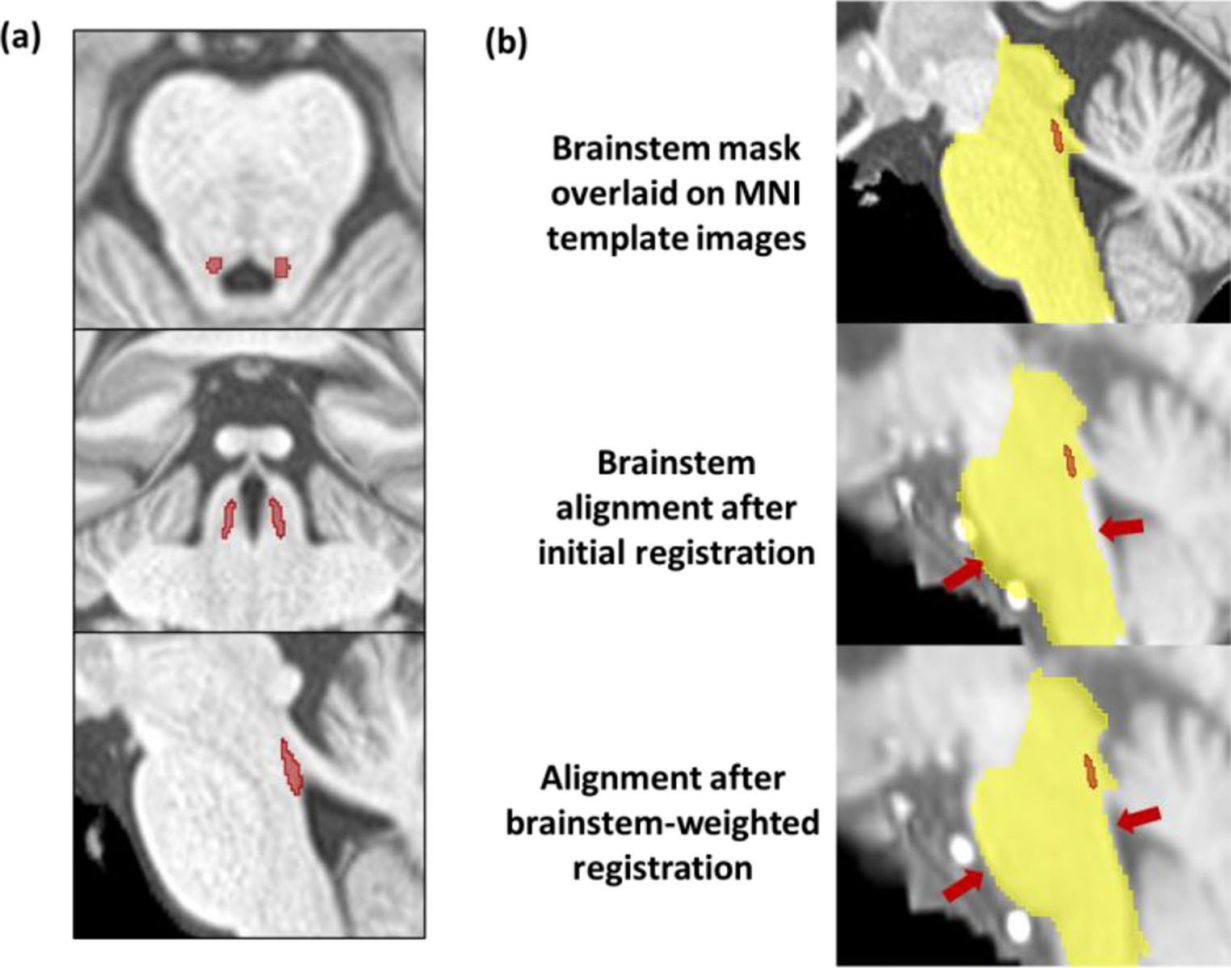
(**a**) The Locus Coeruleus (LC) meta-mask used in this study is overlaid on Montreal Neurological Institute (MNI) template (MNI-ICBM 152 linear space, 0.5 mm resolution) and presented in axial (top), coronal (middle) and sagittal (bottom) view. (**b**) Example of Automated Brainstem Co-registration (ABC) method used in this study. The initial global registration results in misalignment between the brainstem and the reference brainstem mask (yellow), while the subsequent brainstem-weighted registration corrects these misalignments, enabling accurate localization of the LC

Fig. 2a illustrates the identification of LC structure using the meta-mask, overlaid on the MNI atlas. Fig. 2b illustrates registration steps implemented in this study. The ABC method begins with an initial registration stage that aligns global features of individual SPGR images to the MNI atlas. While this step may result in misalignment of the brainstem, the subsequent brainstem-weighted registration refines the alignment, accurately matching individual brainstems to the reference.

**Fig. 3:**
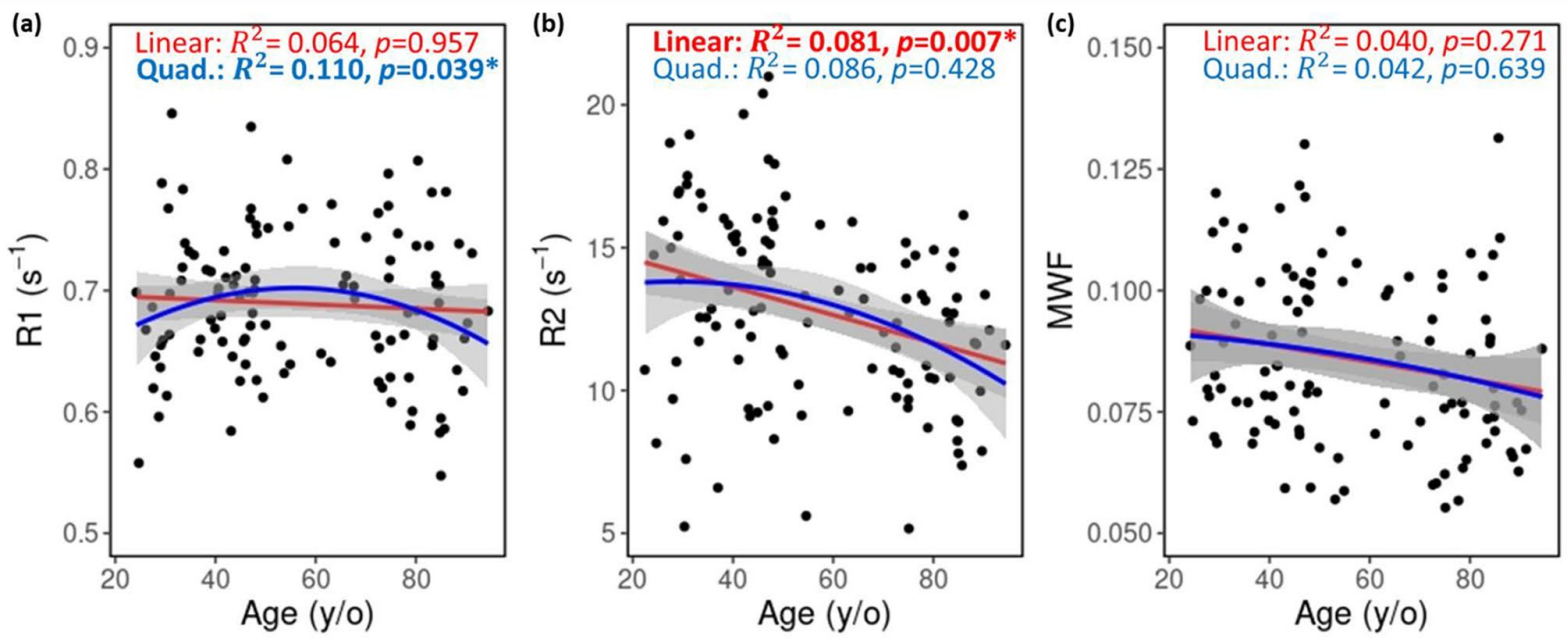
Example of associations between LC quantitative MRI (qMRI) metrics and age, adjusted for sex, race, and years of education. Both linear (red) and quadratic (blue) models were tested. LC-R₁ showed a significant correlation with age^2^, whereas LC-R₂ exhibited a significant negative correlation with age

Fig. 3 shows quantitative results for the qMRI measures (R_1_, R_2_, and MWF), averaged over the entire LC region, plotted as a function of age across all participants. LC-R_1_ exhibited a significant quadratic relationship with age, whereas LC-R_2_ showed a significant linear decline with increasing age. LC-MWF exhibited no significant association with age

**Fig. 4:**
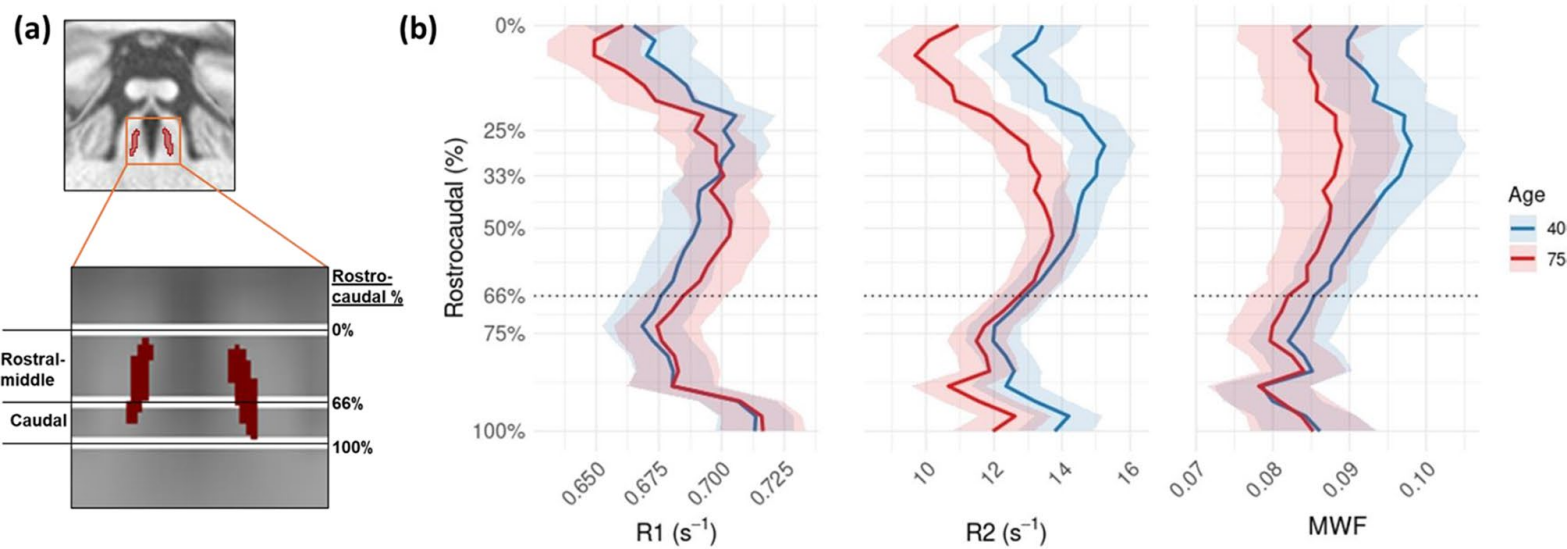
(**a**) Coronal view of LC structure for rostro-caudal identification. By dividing the LC with equidistant segments, the rostral-middle (0–66% rostro-caudal percentile) and caudal (66–100^%^ rostro-caudal percentile) regions of the LC are delineated. (**b**) Predicted qMRI measures across LC slices for individuals aged 40 and 75. Although statistical significance for age was observed only in the rostral and caudal R_2_ values, all three metrics exhibited more pronounced age-related differences in the rostral-middle region of the LC

Fig. 4a shows the segmentation between the rostral-middle (0–66th LC rostro-caudal percentile) and caudal regions (66 − 100th LC rostro-caudal percentile). Fig. 4b illustrates predicted qMRI values at each slice level for ages 40 and 75. All three metrics demonstrated more pronounced age-related differences in the rostral-middle regions, as compared to the caudal region. Statistically, only R_2_ values demonstrated a significant effect of age (*p* < 0.05), specifically in the rostral (0–44th LC rostro-caudal percentile) and restricted caudal (86–100th LC rostro-caudal percentile) regions of the LC.

**Fig. 5:**
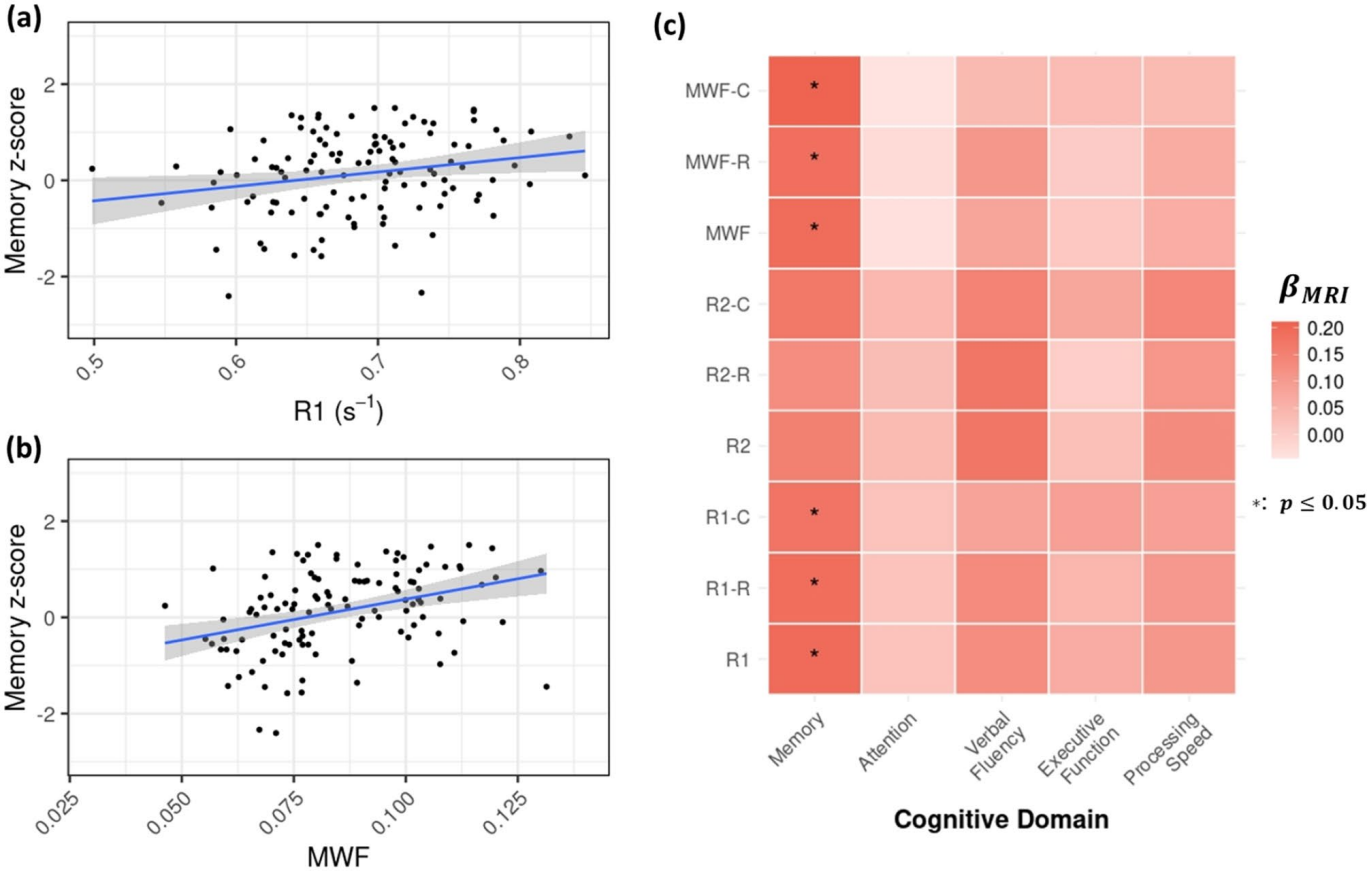
Significant positive associations were observed between cross-sectional memory scores and both (**a**) R_1_ and (**b**) MWF. (**c**) Heatmap displaying color-coded Beta coefficients representing associations between quantitative MRI (qMRI) metrics and cross-sectional cognitive measures. Notably, R_1_ and MWF values in both the rostral-middle and caudal LC showed significant positive associations with memory performance. Reported p-values are FDR corrected. (qMRI-R: qMRI measures in the rostral-middle LC, qMRI-C: qMRI measures in the caudal LC)

As shown in Fig. 5(a-b), our cross-sectional analysis revealed that both LC-R_1_ and LC-MWF values showed a significant positive correlation with memory scores ($\:{p}_{corr}$<0.05). Further, as depicted in Fig. 5c, subregional analysis revealed that R_1_ and MWF values in both rostral-middle and caudal LC were positively associated with memory performance ($\:{p}_{corr}$<0.05).

**Fig. 6:**
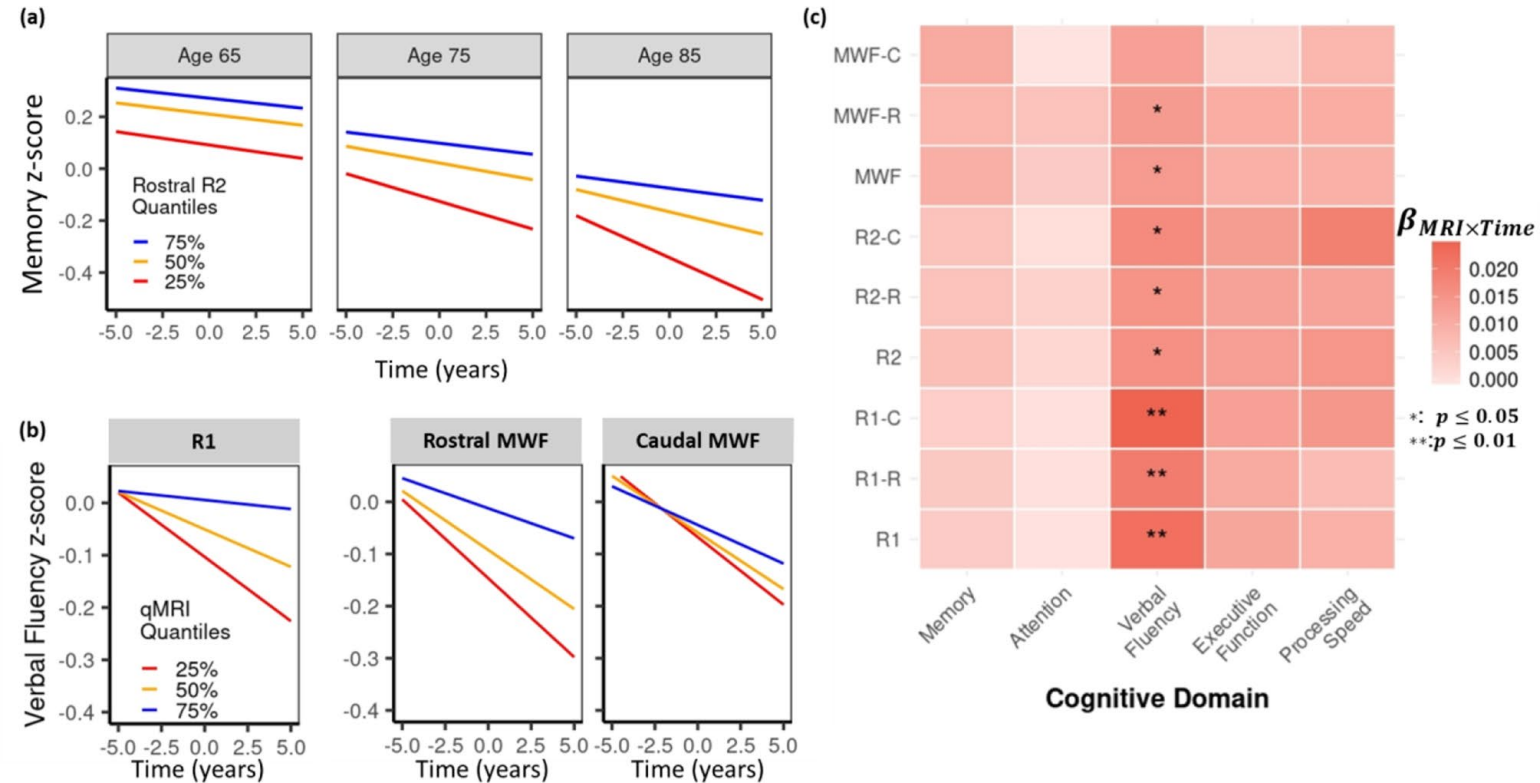
(**a**) Predicted longitudinal trajectories of memory performance illustrating the age-moderated effect of Rostral-R_2_. Lower Rostral-R_2_ values (red lines) are associated with steeper decline in memory, particularly at advanced age. (**b**) Lower R_1_ and R_2_ values in both LC subregions are significantly associated with changes in verbal fluency. MWF values in the rostral, but not caudal, showed a significant association with changes in verbal fluency, as reflected in the distinct trajectories predicted by Rostral-MWF. (**c**) A heatmap illustrating the resulting coefficient for MRI×Time interaction term from the linear mixed effects models across cognitive domains. Each MRI metric was mean-centered and resulting p-values were adjusted for False Discovery Rate correction. Time on the x-axis represents the relative number of years from the MRI scan, where Time = 0 marks the time of the scan

Fig. 6(a) demonstrates a significant age-moderated effect of Rostral-R_2_ measures on changes in memory performance ($\:p$ =0.004). Individuals with lower Rostral-R_2_ values exhibited a steeper decline in memory (red lines) compared to those with higher values (blue lines), particularly at older ages. Although this effect did not survive FDR correction, the corrected p-value remained close to the significance threshold ($\:{p}_{corr}$ =0.062). Additionally, R_1_ and R_2_ in both the rostral-middle and caudal regions were strongly associated with changes in verbal fluency. Interestingly, MWF values in the rostral-middle LC, but not in the caudal region, were positively correlated with changes in verbal fluency (Fig. 6b-c). Full model results, including beta coefficients and corrected p-values, are reported in the Supplementary material

Finally, our sensitivity analysis confirmed that the original LC localization accurately targeted the LC. As shown in Figure S1b, qMRI metrics extracted from surrounding tissue eliminated the significant association with memory. Additionally, even minimal shifts of the mask (Figure S1c-d) led to diminished or absent associations. In contrast, our LC localization method using the meta mask resulted in robust associations with memory, consistent with the known functional roles of the LC.

## Discussion

In this study, we leveraged advanced MR relaxometry to assess the microstructural integrity of the LC, and to investigate how age-related changes in the LC microstructure relate to cognitive performance. Consistent with previous studies [8, 33], we observed age-related alterations in R_1_ and R_2_ measures, indicating a decline in structural integrity with advancing age. Our topographical evaluation of the LC integrity reveals prominent reduction of R_2_, reflecting neuronal density, and MWF in the rostral-middle LC as compared to the caudal region. Given that the rostral-middle LC contains noradrenergic neurons projecting to the cortex, hippocampus, thalamic nuclei [62], and is critically involved in memory and learning [63], the observed age-related differences in this region further underscore its potential relevance to cognitive performance. Indeed, our qMRI measures were associated with both cross-sectional cognitive performance and longitudinal declines in cognitions traditionally linked to LC function, highlighting the relevance of LC integrity in normal aging. Our results demonstrate that R_1_ measures in the LC exhibited a significant positive association with cross-sectional memory scores. We conjecture that our R_1_ measures reflect paramagnetic neuromelanin levels, a byproduct of noradrenaline synthesis. In addition to its inverse relationship with tissue water content [32], a marker of structural degeneration, lower R_1_ may also indicate compromised noradrenaline synthesis associated with aging, which in turn contributes to lower memory performance. Although R_1_ measures are known to be sensitive to myelin [32], the rostrocaudal profiles of LC-MWF exhibited distinct patterns from R_1_, suggesting that R_1_ measures may be capturing other biological properties, as discussed above. Additionally, R_2_ measures in the rostral-middle LC showed an age-moderated effect on the rates of memory decline, substantiating the functional importance of this region in regulating memory. Since R_2_ measures are sensitive to neuronal density, we hypothesize that age-related reductions in neuronal density may compound memory decline with advancing age. Recent longitudinal evidence [64] demonstrates that noradrenergic LC and dopaminergic substantia nigra-ventral tegmental area are differentially associated with late-life memory decline, with LC integrity specifically linked to episodic memory. Notably, changes in LC integrity over time predicted future memory performance, complementing our findings by reinforcing the critical role of the noradrenergic system in memory performance. Our study also introduced an additional measure for evaluating microstructural integrity, specifically the myelin contents of the LC. While there are limited studies reporting extremely low, yet detectable levels of myelin are present in the LC, along with poorly myelinated axons from the LC neurons [35], our results indicate that MWF in the LC was significantly associated with cross-sectional memory assessments. Interestingly, such association were present in both the rostral-middle and caudal LC. Further studies are needed to understand whether short T_1_ and T_2_ components captured by MWF measurements represent water trapped in the myelin sheaths, or other potential biological substances exhibiting age-related changes. Another notable observation is the strong relationship between LC integrity and longitudinal changes in verbal fluency. Several studies identify the impact of degraded LC integrity to lower verbal fluency [21, 22]. Indeed, a pharmacological study found that enhancing noradrenergic neurotransmission in the prefrontal cortex of Parkinson’s disease (PD) patients improved verbal fluency performance, highlighting a critical link between noradrenergic system and verbal fluency [65]. Overall, our results highlight the utility of advanced MR relaxometry in capturing region-specific microstructural changes in the LC, particularly in the rostral-middle segment. Our qMRI measures demonstrated strong associations with cognitive functions linked to the noradrenergic system, underscoring the potential of our technique as a quantitative tool for investigating age-related changes in LC structure.

Quantitative assessment of LC integrity is especially important given its early and progressive vulnerability to neurodegenerative processes. Animal studies have shown that tau pathology in the LC leads to neuronal loss and cognitive deficits [66], while post-mortem human brain studies have associated reduced LC volume and increased tau burden with advancing AD [67, 68]. Progressive LC neuronal loss has also been observed in individuals with mild cognitive impairment (MCI) and AD, correlating with cognitive decline [69]. Although preclinical and ex vivo data consistently support a link between LC degeneration and cognitive impairment, in vivo MR imaging studies have reported mixed results. Some studies indicate reduced LC-CR in neurodegenerative and psychiatric conditions (e.g., PD, AD, depression) [26, 70, 71], while others show no significant differences [72]. Additionally, age-related changes in LC-CR further complicate the interpretation. For instance, Olivieri et al. [73] observed significant LC signal reduction in both AD and MCI groups compared to healthy controls, while Betts et al. [74] only observed such a change in the AD group, but not in the MCI group. These discrepancies call into question the sensitivity of the imaging measures used in these studies. Indeed, the commonly used contrast ratio measure may be biased by physiological changes in the reference tissue, which can subsequently bias the LC signal [20]. Furthermore, methodological differences in LC signal estimation, such as computing CR of LC-signal averaged from both left and right [74], using raw signal intensity [73] or localizing the signal to a specific location of LC [75], can also lead to inconsistencies. Additionally, the acquired signal intensity can substantially vary depending on the types of MR sequence, sequence parameters (e.g., flip angle, echo time, repetition time), and hardware (e.g., coil geometry, signal amplifier) [76], preventing direct measurement or comparison among subjects or across studies. Moreover, previous studies often used 2D imaging techniques with thick slice profiles. While thicker slices enhance signal-to-noise ratio, they may hinder accurate identification of the rostro-caudal LC. Further, variations in slice thickness could introduce inconsistencies in the computed LC-CR, further complicating the interpretation of the results. These limitations in using CR as a quantitative metric for LC integrity impair the accurate assessment of neuronal degeneration in the LC and reduce the sensitivity of in vivo, image-derived measurements.

In comparison, qMRI techniques provide imaging biomarkers that are sensitive to tissue microstructures and composition, enabling the assessment of subtle changes in brain tissue. Notably, diffusion tensor imaging (DTI) has been employed to quantitatively evaluate the microstructure of the LC. A post-mortem DTI study revealed increased fractional anisotropy (FA) in the brains of individuals with AD, suggesting a loss of noradrenergic cells and fibers, which is consistent with the neuropathological hallmarks of AD [77]. Additionally, Quanttrini et al. [78] demonstrated increased diffusivity along the LC-transentorhinal cortical pathway, a finding consistent with a stage subsequent to the initial spread of tau pathology in the LC [6]. Furthermore, the sensitivity of qMRI revealed microstructural changes in the LC during normal aging characterized by reduced mean and radial diffusivities and increased FA in older compared to younger individuals [79]. These studies also showed that these changes are significantly correlated with cognitive decline. However, diffusion imaging protocols are typically limited by low spatial resolution, as used in the aforementioned studies. These low-resolution images may hinder the accurate identification of microstructural changes in small structures, such as the LC. Alternatively, MR relaxometry, particularly BMC-mcDESPOT used in our study, provides whole-brain, isotropic high-resolution mapping of relaxation rates, which can serve as a proxy to infer tissue microstructures and composition. In addition, these metrics are less dependent on methodology or hardware compared to signal intensity-based features and have been extensively applied to various neuropathologies [80, 81]. The current study demonstrates that our qMRI measures quantitatively assess LC integrity to capture early and region-specific microstructural alterations relevant to cognitive functioning. These quantitative imaging biomarkers are biologically interpretable, providing valuable insights into unknown mechanisms of brain aging. These biomarkers hold significant promises for detecting LC degeneration at preclinical stages, enabling improved monitoring of disease progression and potentially guiding early intervention strategies in age-related neurodegenerative diseases, such as AD. Future studies expanding the cohort to include patients with clinical AD symptoms will deepen our understanding of how LC integrity contributes to disease progression and underlying pathological mechanisms.

There are limitations to the current study. First, participants were not recruited uniformly across all age intervals in our cohort. The number of participants between 50 and 65 years old was noticeably lower than other ages, which may have hindered the ability to observe potential inverted U-shape trajectories in our findings. While this might impact the interpretability of our findings in LC structural changes during normal aging, we obtained a meaningful sample size across the age range of our study. Furthermore, our cohort had a higher proportion of white participants compared to other racial groups. Future studies with more diverse cohorts are warranted to help identify potential contributions of race-specific genetic predisposition. Despite these limitations, our study has unique strengths. The extensive amount of longitudinal cognitive testing allowed for better characterization of longitudinal decline over long periods, yielding greater confidence in estimating rates of change. Secondly, despite our best effort to accurately align individual images to the template using the ABC methods, some degree of partial volume contamination remains inevitable. Although our sensitivity analyses indicate that the observed signals are most likely attributable to the LC rather than surrounding tissues, future studies employing higher spatial resolution, as well as neuromelanin sensitive imaging protocols are warranted to further minimize partial volume effects and to improve LC localization. Lastly, although we interpreted our findings in the context of physiological features specific to the LC, it is important to note that our qMRI metrics are also sensitive to other biological substances, such as increased water content, myelin, and iron. Furthermore, the underlying mechanisms driving LC contrasts observed in previous studies remain under debate, with many attributing the signal to MT effect [82]. Future studies are needed to validate and clarify the sources of age-related differences in qMRI metrics and LC contrast, to better understand the biological underpinnings of these imaging findings. Also, extending the investigation to other brain regions, such as the raphe nuclei, which are also affected early by tau pathology [83], will further supplement our hypotheses and enrich our interpretation in how changes in LC may relate to broader network-levels deterioration in connected brain regions. Additionally, longitudinal imaging studies will provide more comprehensive understanding of how LC structural changes are evolving during aging process.

## Conclusion

We demonstrated the potential of quantitative MRI biomarkers to investigate age-related differences in the LC integrity and their implication in cognitive dysfunction. Our results indicate that LC-qMRI relaxometry metrics are sensitive to age-related differences and cognitive decline. These quantitative biomarkers may offer a more accurate and reliable assessment of LC degeneration, enabling the monitoring of aging processes and facilitating the development of potential interventions to mitigate age-related diseases.

## Electronic supplementary material

Below is the link to the electronic supplementary material.

Supplementary Material 1

Supplementary Material 2

## Data Availability

The datasets generated and/or analyzed during this study are available upon reasonable request to the corresponding author, contingent upon institutional approval.
