# Supplementary material for: Age-related differences in Rostral-Middle locus coeruleus microstructure: A critical role in cognitive decline revealed by magnetic resonance relaxometry
Source: Alzheimers Res Ther. 2025 Jul 15;17:161. doi: 10.1186/s13195-025-01809-4 (PMC12261599; doi:10.1186/s13195-025-01809-4)
Supplement: Supplementary file 1 — Supplementary Material 1 [file 13195_2025_1809_MOESM1_ESM.docx]

Supplementary Materials

**Figure S1**


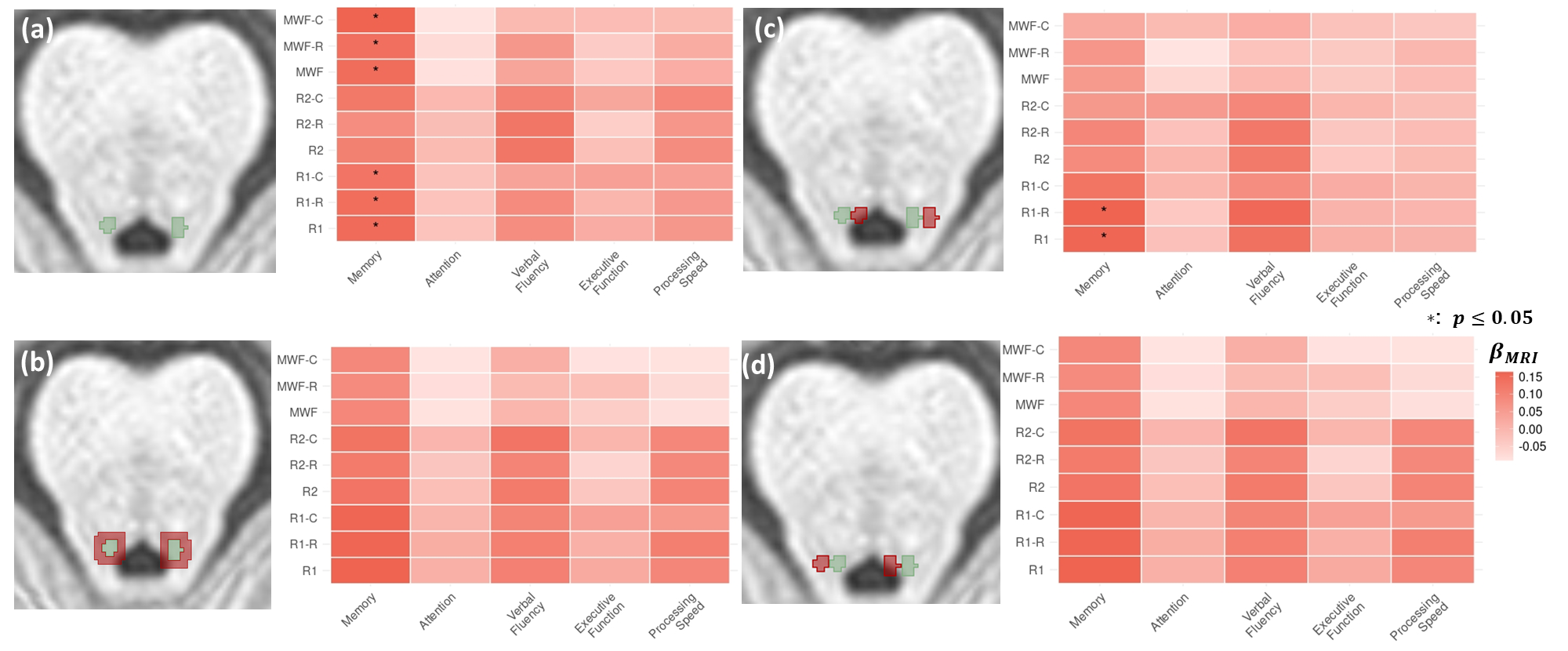


Figure S1. Sensitivity analysis assessing potential partial-volume contamination from surrounding tissues. (a) LC-ROI derived from the meta-mask (green) and corresponding heatmaps illustrating the associations between MRI metrics and memory performance. (b) To sample qMRI metrics from surrounding tissue, the meta-mask is dilated by 3 voxels and LC regions are masked out. This eliminated the significant associations with memory. Additionally, ROIs are deliberately shifted 1.5mm to the (c) right and (d) left, which resulted in reduced or eliminated associations with cognitive performance.

**Table S1.** Multiple linear regression model results for cross-sectional analysis

The model equation is specified as follows:

$${Cog}_{i}= \beta_{0}+\beta_{age}\times{age}_{i}+\beta_{sex}\times{sex}_{i}+\beta_{race}\times{race}_{i}+\beta_{EDY}\times{EDY}_{i}+\beta_{MRI}\times{qMRI}_{i}+\varepsilon_{i},$$

where ${Cog}_{i}$ is the z-scored cognitive domain score at the time of MRI for the *i^th^* participant; ${age}_{i}$, the *i^th^* participant’s age at the time of MRI; ${sex}_{i}$, ${race}_{i}$, and ${EDY}_{i}$ are sex, race, and years of education of the *i^th^* participant; $q{MRI}_{i}$ is qMRI metrics (R_1,_ R_2_ or MWF in whole-LC, Rostral-middle, and Caudal LC); and $\varepsilon_{i}$ is the residual error. For a detailed summary of the model results, please refer to the *Model_Summary_Results.xlsx* spreadsheet, specifically the tabs labeled “Cross-sectional”.

**Table S2.** Linear mixed effects model results for longitudinal analysis

The model equation is specified as follows:

${Cog}_{ij}= \beta_{0}+\beta_{age}\times{age}_{i}+\beta_{sex}\times{sex}_{i}+\beta_{race}\times{race}_{i}+\beta_{EDY}\times{EDY}_{i}+\beta_{time}\times{time}_{ij}+\beta_{qMRI}\times{qMRI}_{i}+\beta_{age\times time}\times{age}_{i}\times{time}_{ij}+\beta_{qMRI\times age}\times{qMRI}_{i}\times{age}_{i}+\beta_{qMRI\times time}\times{qMRI}_{i}\times{time}_{ij}+\beta_{qMRI\times time\times age}\times{qMRI}_{i}\times{time}_{ij}\times{age}_{i}+b_{i}+\varepsilon_{ij}$,

where ${Cog}_{ij}$ is the cognitive score of subjects $i$ at time $j$, ${time}_{ij}$ is the time from MRI of the *i^th^* participant at time point $j$; ${age}_{i}$, the *i^th^* participant’s age at the time of MRI; ${sex}_{i}$, ${race}_{i}$, and ${EDY}_{i}$ are sex, race, and years of education of the *i^th^* participant; $q{MRI}_{i}$ is qMRI metrics (R_1,_ R_2_ or MWF in whole-LC, Rostral-middle, and Caudal LC); ${age}_{i}\times{time}_{ij}$ is the interaction term representing age-moderated changes in cognition; ${qMRI}_{i}\times{age}_{i}$ describes age-moderated cross-sectional effects; $q{MRI}_{i}\times{time}_{ij}$ represents the expectation of the difference in the annual change in cognition per unit difference in qMRI measures; ${qMRI}_{i}\times{time}_{ij}\times{age}_{i}$, captures age-moderated cognitive trajectories by qMRI measures; $b_{i}$ is the random intercept; and $\varepsilon_{ij}$ is the residual error. For a detailed summary of the model results, please refer to the *Model_Summary_Results.xlsx* spreadsheet, specifically the tabs labeled “Longitudinal”.
